# Recent advances of biomaterials in biotherapy

**DOI:** 10.1093/rb/rbw007

**Published:** 2016-03-05

**Authors:** Ling Li, Zhi-Yao He, Xia-Wei Wei, Yu-Quan Wei

**Affiliations:** Laboratory of Aging Research and Nanotoxicology, State Key Laboratory of Biotherapy and Cancer Center, West China Hospital, Sichuan University, Chengdu, Sichuan, 610041, People’s Republic of China

**Keywords:** biomaterials, biotherapy, gene delivery, immunotherapy, tissue engineering

## Abstract

Biotherapy mainly refers to the intervention and the treatment of major diseases with biotechnologies or bio-drugs, which include gene therapy, immunotherapy (vaccines and antibodies), bone marrow transplantation and stem-cell therapy. In recent years, numerous biomaterials have emerged and were utilized in the field of biotherapy due to their biocompatibility and biodegradability. Generally, biomaterials can be classified into natural or synthetic polymers according to their source, both of which have attracted much attention. Notably, biomaterials-based non-viral gene delivery vectors in gene therapy are undergoing rapid development with the emergence of surface-modified or functionalized materials. In immunotherapy, biomaterials appear to be attractive means for enhancing the delivery efficacy and the potency of vaccines. Additionally, hydrogels and scaffolds are ideal candidates in stem-cell therapy and tissue engineering. In this review, we present an introduction of biomaterials used in above biotherapy, including gene therapy, immunotherapy, stem-cell therapy and tissue engineering. We also highlighted the biomaterials which have already entered the clinical evaluation

## Introduction

In recent years, biotherapy has gained more and more attention in the potential treatment of many critical diseases (such as cancer, cardiovascular disease, Aids etc.) [1–4]. Biotherapy connects basic studies and clinical applications, and contributes to the development of bench to bedside translation [5–7]. Generally, biotherapy mainly refers to the intervention and the treatment of critical diseases with biotechnologies or bio-drugs, including the discovery of therapeutic targets by pathology investigation and the development of biotechnologies associated with gene therapy, immunotherapy, stem-cell therapy and tissue engineering (bone, heart, liver etc*.)* [8, 9]. Gene therapy is the intentional modulation of gene expression in specific cells or tissues to treat pathological conditions and immunotherapy can be summarized as the treatment of disease by regulating immune responses, mainly by vaccines and antibodies [10, 11]. Efficient and targeted delivery of antigens, immunomodulatory or immunostimulatory molecule to the appropriate cell is critical for an efficient immunotherapy. Stem-cell therapy is to use stem cells to treat or prevent diseases or condition, which has been widely applied in the treatment of hematological diseases, cancers, cardiovascular and cerebrovascular diseases [12–15]. In addition, bone marrow transplant is one of the most widely used stem-cell therapy. Tissue engineering is a newly emerging biomedical technology, which aids and increases the repair and regeneration of deficient and injured tissues.

Currently, many significant achievements have been made in the biotherapies for the treatment of some critical diseases. In the meantime, biomaterials have attracted much attention in biotherapy, including various fields such as regenerative medicine, gene delivery, stem-cell therapy, tissue engineering and immunomodulation [16–19]. Biomaterials are generally classified as two groups, natural and synthetic based on their origin. Natural biomaterials, such as hyaluronic acid, alginate, chitosan, heparin and gelatin, seem to be attractive due to their excellent biocompatibility and have been used for a long time [20–23]. Meanwhile, with the emergence of large amounts of synthetic biodegradable polymers, synthetic polymers have gained significant attention due to the characteristics of easy manipulation, large-scale production [19]. In this review, we address the biomaterials which have already been used or with the potential applications in biotherapy including gene delivery, immunotherapy, stem-cell therapy and tissue engineering. In addition, the clinical trials of those biomaterials in biotherapy are highlighted.

## Biomaterials in gene therapy

Over the past two decades, gene therapy has gained significant attention for the treatment of many inherited diseases and genetic disorders [24, 25]. Safe and effective gene delivery systems are urgently needed for enhancing the efficiency of gene therapies [26, 27]. Although many viral vectors, including adenovirus, adeno-associated virus, lentivirus and retrovirus have been widely investigated in gene delivery, severe immune/inflammatory reactions, the risk of recombination with wild-type viruses, limited cargo packaging capacity and difficulty of production significantly limited their further application [28–30]. In recent years, biomaterials-based non-viral gene delivery vectors, including cationic polymers, lipids, dendrimers and peptides have been proposed as alternatives for gene delivery, largely attributed to their low immunogenecity, the absence of endogenous virus recombination, construction flexibility and facile fabrication [31–33]. More importantly, some of these non-viral gene vectors have successfully entered the clinical trials. In this section, we summarize most commonly used non-viral gene vectors and highlight their applications.

### Lipid-based gene vectors

Lipid-mediated gene transfer was one of the earliest strategies in gene therapy. Many types of cationic lipids, including 1,2-dioleoyl-3-trimethylammoniumpropane (DOTAP), N-[1-(2,3-dioleyloxy)propyl]-N, N, N-trimethyl-ammonium chloride, and 1,2-dimyristyloxypropyl-3-dimethyl-hydroxyethyl ammonium bromide (DMRIE) are commercially available. As the most classical non-viral gene vectors, cationic liposomes are the first non-viral delivery vectors used in clinical trials [34]. Nowadays, many lipid-based gene transfection reagents are commercially available, such as Lipofectamine 2000, Lipofectamine 3000 and Lipofecter etc. However, the drawbacks of poor stability, low transfection efficacy and the generation of inflammatory response have limited the application of cationic lipids-based nanocarriers to some extent [35]. Many explorations have been carried out to promote gene transfection efficacy and reduce the cytotoxicity of cationic lipids-based nanocarriers. Among them, manipulation of cationic head group, varying the lengths and types of hydrophobic tail group has been widely investigated. In addition, in our group, the modification of carrier surface has been carried out and folate-linked lipoplexes for targeted delivery of shRNA in ovarian cancer has achieved great efficacy [36].

Promisingly, some cases of liposome-mediated gene delivery for the treatment of genetic and metabolic disorders or cancers have been evaluated in the clinical stage. A Phase I pilot study of gene therapy for cystic fibrosis using cationic liposome (DMRIE/DOPE) mediated gene transfer has been completed (NCT00004471). Another Phase I clinical research study of DOTAP-Chol-Fus1 liposome-mediated gene therapy for the treatment of non-small cell lung cancer and small cell lung cancer has been completed as well (NCT00059605). Meanwhile, Another Phase I trial of intratumoral epidermal growth factor receptor antisense DNA delivered by DC-Chol liposomes in advanced head and neck cancer including oral squamous cell carcinoma (NCT00009841) were conducted, respectively by University of Pittsburgh Cancer Institute. Additionally, in a Phase I study, DC-Chol/DOPE cationic liposomes were employed to deliver the human HLA-A2, HLA-B13 and the murine H-2K genes to patients with different cancer types. Strong immune response was generated locally following *in situ* gene therapy with no significant side-effects. Two out of eight patients showed the complete regression in cutaneous nodules after HLA-A2-DNA liposome treatment [37]. Other liposomal formulations under clinical investigation include GL67A-DOPE-DMPE-poly(ethylene glycol) (PEG), DOTAP-cholesterol and GAP-DMORIE-DPyPE (NCT01621867; NCT00059605; NCT01502358).

### PLL-based gene vectors

Poly(L-Lysine) (PLL) is one of the most widely studied gene carriers with effective gene condense activity. PLL is a synthetic polypeptide with good biocompatibility and biodegradability. However, PLL exhibits relatively low transfection efficiency attributed to the lack of buffering capacity to aid in endosomal escape. In order to promote the gene transfection efficacy of PLL, many approaches have been investigated. Among them, incorporation hydrophobic moieties into the PLL carriers could be the most widely used approach. For instance, Incani *et al.* constructed amphiphilic lipopolymer by substituting the endogenous palmitic acid for PLL. The transfection efficiency was significantly increased, even higher than the commercial transfection agent Lipofectamine 2000 [38]. Meanwhile, to promote the endosomal escape ability of PLL, histidine was conjugated to PLL [39]. In addition, hydrophilic PEG has been conjugated to PLL to decrease the interaction with serum proteins and extend the circulation time in the bloodstream [40]. Furthermore, to achieve the targeted gene delivery *in vivo* to minimize the side effects, specific targeting moieties, such as antibodies, peptides and folate, have been introduced to PLL. Kataoka *et al.* modified PEG-PLL with the cyclo-arginine-glycine-glutamic acid (cRGD) peptide, which binds to integrin αvβ3 receptor over expressed on tumor neovasculature and many types of tumors. It was observed that the incorporation of cRGD peptide into PEG-PLL resulted in increased gene silencing ability *in vitro* and improved accumulation in both the tumor mass and tumor-associated blood vessels after systemic administration [41].

### Polyethylenimine-based gene vectors

In the past decades, polyethylenimine (PEI) has been considered as a gold standard for polymer-based gene transfection since its introduction in 1995 [42]. The success of PEI for gene transfection is primarily attributed to its high charge density and endosomal escape ability. PEIs primarily have two different topologies (linear and branched structures) and are available in a wide range of molecular weights (MWs), ranging from 423 Da to 800 kDa [43]. Both the efficacy and toxicity of PEI are strongly correlated with its Mw and structure. Generally, PEIs with a high branching degree, high molar mass and high cationic charge densities exhibit high gene transfection efficiency while significant cytotoxicity as well. However, low MW PEIs, are less cytotoxic but also less efficient [44]. An extensive variety of modifications have been employed to improve the transfection efficiency and decrease the cytotoxicity of the PEI polymer. Modification PEI with hydrophobic moieties, such as lipids, cholesterol, stearic acid or palmitic acid has been validated as successful approaches [45–47]. In addition, PEG is introduced to PEI to promote the stability of PEI-based polyplexes in physiological conditions. PEGylation creates a hydrophilic shell for PEI, avoid the exposure of positive surface charge and stabilize the resultant polyplexes in physiological buffers by reducing non-specific interactions with serum proteins [48]. Furthermore, targeting moiety, such as folate, RGD peptide and galactose were also introduced into PEI to attenuate the ‘PEG dilemma’ and achieve the targeted gene delivery *in vivo* [49, 50]. Additionally, introducing stimuli-responsive linkages (reducible disulfide linkages or ester conjugation) to cross-link low MW PEI is also an alternative method to achieve high transfection efficiency while maintaining cell viability [51, 52].

Currently, PEI is involved in several clinical trials. One example is gene therapy product CYL-02 (plasmid DNA pre-complexed to linear PEI encoding sst2+ dck::umk genes), which has completed its Phase 1 clinical trials for the treatment of unresectable pancreatic carcinoma (NCT01274455). DTA-H19/PEI has successfully moved to its Phase 2 clinical trials (NCT00595088) for the treatment of intermediate-risk superficial bladder cancer. The clinical trials with SNS01-T in the treatment of relapsed or refractory B cell malignancies (Multiple Myeloma, B-Cell Lymphoma or Plasma Cell Leukemia) are ongoing (NCT01435720). Additionally, EGEN-001, an IL-12 plasmid formulated with PEG-PEI-cholesterol lipopolymer, is under Phase II clinical investigation for the treatment of persistent or recurrent epithelial ovarian, fallopian tube or primary peritoneal cancer (NCT01489371, NCT01118052, NCT01300858) [53].

### Polyamidoamine dendrimers-based gene vectors

Polyamidoamine (PAMAM) dendrimers have become the most utilized dendrimer-based vectors for gene transfer due to ease of synthesis and commercial availability. Commonly, PAMAM dendrimers possess generation-dependent properties. Low generation PAMAM dendrimers, such as G0-G3 exhibit poor gene transfection efficiencies and less cytotoxicities, while high generation, such as G4–G8 show better gene transfection efficiencies but certain cytotoxicities [54]. As the same with PLL and PEI polymers, various alterations to the pristine PAMAM dendrimer structure have been tested to improve the transfection efficiency and reduce cytotoxicity [55–57].

### Chitosan-based gene vectors

Chitosan has become one of the most prominent, naturally derived non-viral vectors for gene transfer due to its biodegradability and biocompatibility. However, its application in gene delivery is significantly limited by relatively low transfection efficiency. In previous study, stearic acid, deoxycholic acid, 5β-cholanic acid and hydrophobic alkyl chains have been conjugated to chitosan to improve the cellular uptake. For instance, Kwon *et al.* [58] modified glycol chitosan with 5β-cholanic acid to promote gene delivery. The modified complexes showed increasing transfection efficiencies both *in vitro* and *in vivo*. Meanwhile, to improve the buffering capacity of chitosan-based polyplexes, imidazole or PEI, which has ‘proton sponge’ effect, was conjugated to chitosan. Furthermore, various cell/tissue-targeting ligands were introduced to chitosan to achieve the targeted gene delivery [59, 60].

Other non-viral gene vectors are currently being evaluated pre-clinically for gene delivery, such as Poly[2-(dimethylamino) ethyl methacrylate], poly(β-amino ester)s, poly(amidoamine)s and various carbohydrate-based polymers and dendrimers [61–64].

## Biomaterials in immunotherapy

Immune system plays a critical role in the health of organisms and can be either a cure or cause of diseases. Strategies to enhance, suppress or qualitatively shape the immune response are of great importance for diverse biomedical applications, such as the development of new vaccines, treatments for autoimmune diseases and allergies, immunotherapies for cancer and strategies for regenerative medicine. Currently, increasing interests are focusing on engineering biomaterials to rationally control the immune system by enhancing or suppressing immune reactions in an antigen-specific or nonspecific manner to treat disease or overcome adverse immune situations. Among them, new strategies for vaccination using biomaterials are highlighted.

Vaccination is an important way of controlling and potentially eliminating infectious diseases and cancers. Traditional live-attenuated vaccines have been used for a long time, but serious safety concerns regarding toxicity and the risk of mutation back to the infectious pathogen have largely limited their application [65, 66]. In recent years, subunit vaccines composed of purified or recombinant antigens with ensure safety have gained much attention, but they do not provide sufficient immunostimulation necessary for robust protection [67, 68]. There remains a big challenge to elicit potent antibody production and CD8^+ ^T cell response. Biomaterials-based antigen delivery systems have emerged as an innovative strategy to improve the efficacy of subunit vaccines. The antigen delivery systems are often used to enhance the delivery and presentation of antigens to antigen presenting cells (APCs) in order to improve the efficacy of the vaccination strategy.

The antigen delivery systems are usually roughly classified into two categories: one is particulate-based delivery systems (microparticles, nanoparticles), the other is hydrogel and scaffolds-based delivery systems (solid implants/scaffolds, hydrogels) [69, 70]. Pioneering work has been done in encapsulating antigens, immunomodulatory agents and immunostimulatory drugs inside antigen delivery systems. Meanwhile, the delivery systems may exert different functions, depending on the specific properties of delivery system. This includes stabilizing, protecting the antigen from degradation and delaying the clearance of antigen from the injection site, through sustaining the release of the antigen and/or by providing a depot at the injection site. Additionally, delivery systems can targeting delivery the antigen to the desired subset of APCs and facilitate the antigen uptake and/or the intracellular trafficking and antigen release in APCs [71, 72].

### Lipid-based particulate delivery system

Lipid-based delivery systems like emulsions, virosomes and liposomes have been widely used in vaccines, even some of them like liposomes and ISCOMs are undergoing clinical development [73]. Liposomes are small artificial vesicles with spherical shape formed by amphiphilic phospholipids (soybean phosphatidylcholine (SPC), phosphatidylethanolamine (PE), distearoyl phosphatidylethanolamine (DSPE), DOTAP etc.) and cholesterol which self-assembled into one or more bilayers enclosing an aqueous core. They are versatile antigen delivery systems since their physicochemical properties can be easily varied by adjusting the lipid composition and the content of additional immunopotentiating components that associated, encapsulated or intercalated in the lipid membrane [72].

Cationic liposomes based on dimethyldioctadecylammonium and the immunopotentiating glycolipid trehalosedibehenate (Adjuvant CAF01) can promote humoral immune responses and cell-mediated immune responses in preclinical animal models and has completed its Phase I clinical trial in combination with HIV-1 peptides for treatment of patients with chronic HIV-infection (NCT01009762). Another example is ISCOMs, which are lipid particles comprising cholesterol, phospholipids and cell membrane antigens with the immunostimulatory fraction from Quillaja saponaria (Quil A) incorporated. ISCOMs have been shown to induce both humoral and cellular immune responses in humans and evaluated in clinical trials [72]. Additionally, Tecemotide, a therapeutic vaccine consists of a MUC1 lipopeptide combined with monophosphoryl lipid A in a liposomal delivery vehicle, has been designed to induce immune response to cancer cells expressing MUC1. In the Phase III START trial, in a preplanned subgroup analysis for stratification variables, Tecemotide improved survival in patients who had received concurrent chemoradiotherapy (30.8 versus 20.6 months; HR 0.78, *P* = 0.016) [74, 75]. In another study, liposome-based vaccines containing an extract of a person’s cancer cells and the immunostimulant interleukin-2 has been moved to the Phase 1 clinical trials for treating patients with previously untreated chronic lymphocytic leukemia (NCT01976520).

### Polymer-based particulate delivery system

Polymeric micro-and nanoparticles have also been studied widely as vaccine delivery systems due to their ability to mediate cross-presentation [76]. The most commonly used polymer for vaccination is the biocompatible and biodegradable polymer poly(lactic-co-glycolic acid) (PLGA) formulated into either nano- or microparticles. PLGA microparticles encapsulating antigen have been shown to enhance cross-presentation and the induction of CTL responses [77]. Particularly, combination PLGA particles with immunopotentiating compounds have been validated to be a promising strategy for further improving the vaccine efficacy [72, 78].

Polyelectrolyte capsules fabricated by layer-by-layer (LBL) coating of template nano- or microparticles with oppositely charged polyelectrolytes have also been applied to encapsulated protein or peptide antigens [79]. It has been demonstrated that LBL-assembled disulfide cross-linked poly(methacrylic acid) (PMA_SH_) hydrogel capsules can improve intracellular antigen release and induced CD8+ T cell proliferation in mice towards the encapsulated ovalbumin (OVA) antigen [80].

### Hydrogels and scaffolds-based delivery systems

Polymeric scaffolds and hydrogels with 3D polymeric networks have been widely used for cell encapsulation and controlled release of therapeutic proteins, peptides, drugs and nucleic acids due to their biocompatibility, design flexibility and a broad spectrum of choice of base material [70]. Choice of materials for forming scaffolds and hydrogels ranged from natural polymers (dextran, alginate, gelatin, hyaluronic acid etc.) and entirely synthetic polymers [poly (caprolactone) (PCL), PEG-PCL-PEG, PLGA]. On one hand, 3D scaffolds or hydrogels can be used as immunological microenvironments and delivery of *ex vivo* programmed immune cells, such as dendritic cells (DCs) and adoptive T cells, attributed to their macroporous properties. On the other hand, they can also been used to simultaneously encapsulate antigen and adjuvant and provide a depot to controlled release the loading cargos, which could induce and program immune cells *in situ*.

Especially, the breakthrough in scaffold-based cancer immunotherapy is the development of ‘WDVAX’ vaccine, which has been advanced to Phase I clinical trial for treatment of melanoma (NCT01753089). ‘WDVAX’ vaccine is a tablet-shaped macroporous PLGA scaffold containing tumor lystaes (antigen) with two immune stimulatory proteins (granulocyte-macrophage colony-stimulating factor (GM-CSF), immunostimulatory CpG oligonucleotide). In the preclinical study, these scaffolds recruited naive DCs and programmed them to induce robust prophylactic immunity against murine B16F10 melanoma tumor. Their studies showed that GM-CSF enhanced DC recruitment to the implantation site and residing inside the implant. Subsequently, CpG oligonucleotides (complexed to cationic polymer PEI) loaded into the scaffolds activated the DC in the implant *in situ* [81]. More importantly, the group of David Mooney also investigated the ability of these scaffolds to provide therapeutic vaccination against established melanoma. The researchers combined this vaccine with antibodies that block either the protein cytotoxic T lymphocyte antigen4 or a protein called programed death 1, two immune checkpoint receptors. They found a single dose of the vaccine alone inhibited cancer growth in mice with established melanoma. Taken together, these studies showed that by using polymeric scaffolds delivering multiple immunomodulatory molecules could stimulate CD4+ and CD8+ cellular response.

## Biomaterials in stem-cell therapy and tissue engineering

Stem-cell-based therapies have existed since the first successful bone marrow transplantations in 1968 [82, 83]. In the subsequent development, significant progress has been made in the field of cardiovascular disease, tissue engineering (cartilage, bone, spinal injury etc.) and cancers by stem-cell-based therapies. For instances, transplantation of stem cells into the heart can improve cardiac function after myocardial infarction and in chronic heart failure [84]. Mesenchymal stem-cell therapy is capable to rebuild cartilage [85]. However, survival of transplanted stem cells into diseased tissues or organs is poor, and new strategies are needed to enhance stem cell differentiation and survival *in vivo*. In recent years, progress in biomaterials design and engineering enabled a new generation of instructive materials to emerge as good candidates for stem-cell-based therapies [86]. On one hand, biomaterials, which mimic naturally occurring extracellular matrix (ECM) could instruct stem cell function in different ways. On the other hand, biomaterials could also able to promote angiogenesis, enhance engraftment and differentiation of stem cells, and accelerate electromechanical integration of transplanted stem cells. Additionally, proteins (growth factors), genes, or drugs can also be delivered together with stem cells through biomaterials. Many types of natural and synthetic biomaterials have been applied to deliver stem cells, such as collagen, fibrin, matrigel, alginate, silk fibroin, PLGA etc [87–91]. Because there have been many reviews concerned about the applications of biomaterials in stem-cell therapy [84, 92–94], so in this section, we just focus on those materials in the clinical trials for stem-cell therapy.

Recently, a filler agent composed of mesenchymal stem cells obtained from autologous adipose tissue associated with hyaluronic acid has moved to their Phase 1 clinical study (NCT02034786). More recently, tissue engineering based on the use of mononuclear cells from autologous bone marrow seeded on porous tricalcium phosphate (TCP) biomaterial to treat patients with pseudoarthrosis have completed its Phase 2 clinical trials (NCT01813188). Meanwhile, a phase II clinical trial to assess the effect of HC-SVT-1001 (autologous fat stem adult mesenchymal cells expanded and combined with a TCP biomaterial) in the surgical treatment of atrophic pseudarthrosis of long bones is currently recruiting participants (NCT02483364). Additionally, a clinical trial based on mesenchymal stem cells from deciduous dental pulp associated with a collagen and hydroxyapatite biomaterial (Geistlich Bio-Oss) to reconstruct the alveolar bone defect in cleft lip and palate patients is ongoing (NCT01932164).

In the past decades, treatment of injured tissues or organs focused on the use of autologous and allogenic grafts [95]. However, these practices had significant limitations. Autologous grafts may cause donor site morbidity and consequent loss of organ functionality, while allografts are associated with risk of disease transmission and require the use of immunosuppressants with associated side effects [96–98]. There is a critical clinical need for improved therapies to aid in the repair and regeneration of damaged tissues or organs. In recent years, there has been increasing importance on materials that could be used in biomedical tissue engineering. Biomaterials intended for tissue engineering applications target to develop artificial materials which can be used to renovate or restore function of diseased or traumatized tissues in the body [99]. Particularly, tissue engineering-based regenerative medicine strategies are emerging as promising therapeutic modalities, which apply a combination of cells, scaffolds and bioactive factors to restore, maintain or improve the tissue structure and function [100]. However, new biocompatible and non-toxic biomaterials for the manufacture of a new generation of scaffolds comprising adequate mechanical and structural support and able to control cell attachment, migration, proliferation and differentiation are also remains challenging.

In recent years, polymers-based hydrogels or scaffolds have been widely used as biomaterials for the fabrication of medical device and tissue engineering due to their unique properties such as high surface-to-volume ratio, high porosity, biodegradation and mechanical property [99]. Natural polymers such as collagen, chitosan, hyaluronic acid and chondroitin sulfate with low immunogenicity, could be formulated as hydrogel and has been widely applied in soft tissue engineering owing to their low mechanical property [101–106]. Synthetic polymers have an additional advantage due to their tailorable biodegradation rates, higher predictability of properties, easy fabrication and increased mechanical property [107]. Numerous synthetic polymers have been used to fabricate scaffolds including PCL, PLGA, poly-l-lactic acid and poly-glycolic acid. More importantly, these natural or synthetic polymers-based hydrogels or scaffolds can be also used to deliver biomolecules facilitating tissue engineering, such as growth factors (bone morphogenetic protein (BMP), insulin-like growth factor (IGF), fibroblast growth factor (FGF) and vascular endothelial growth factor (VEGF)). These growth factors control osteogenesis, bone tissue regeneration and ECM formation via recruiting and differentiating osteoprogenitor cells to specific lineages [108, 109].

Generally, naturally derived biomaterials are characterized with biocompatibility, enabling the adhesion and migration of cells within their structures. For instance, collagen sponges have long been used to deliver growth factors to promote bone regeneration. Products such as InFuse from Medtronic are approved by FDA for the delivery of recombinant human bone morphogenetic protein-2 (rhBMP-2) through a purified collagen matrix for interbody fusion in the anterior lumbar [110, 111]. Besides naturally derived biomaterials, some synthetic polymers-based scaffolds have been commercially available for tissue engineering. One example is Resomer made using poly(d, l-lactic acid) (Resomer R104, Resomer R203, Resomer R207) and poly(d, l-lactide-co-glycolide) (Resomer RG502), which has been used as bioresorbable implant material. Biodegradable polyesterurethane-foam (DegraPol foam) was also widely applied in tissue engineering. Additionally, PLGA is being extensively investigated for their potential in tissue regeneration, drug and protein delivery which is proven successful by CYTOPLAST Resorb and LUPRON DEPOT. Recently, a PCL/TCP orbital-based implant has advanced into clinical trials for exploring its efficacy in reconstruction of the Orbital walls (NCT01119144). More recently, Pre-cured CPC/rhBMP-2 micro-scaffolds have completed their pilot clinical study for bone regeneration (NCT02609074). In addition, another clinical investigation based on a novel tissue-derived biomaterial to improve soft tissue reconstruction is ongoing (NCT01992315). Very recently, a multicenter clinical trial to evaluate the safety and feasibility of an allogeneic tissue engineered drug (nanostructured artificial human cornea) in patients with corneal trophic ulcers refractory to conventional treatment is currently recruiting participants (NCT01765244).

## Conclusions and perspectives

In the past decades, biodegradable polymers have been widely used in biotherapy because of their biocompatibility and biodegradability. However, the development of biotechnology and medical technology has set higher qualifications for biomedical materials. Novel biodegradable polymers with specific properties are in great demand. When compared with natural biodegradable polymers, synthetic biodegradable polymers have less immunogenicity and easier to be chemically modified and functionalized, which may play more important roles in biotherapy in the future. Moreover, the advancement and rapid translation of recent experimental biomaterials into clinical trials also helps to promote the development of new biomaterials, which might contribute to the development of efficient therapy in related diseases.
